# MicroRNA and Male Infertility: A Potential for Diagnosis

**Published:** 2014-07-08

**Authors:** Yahya Khazaie, Mohammad Hossein Nasr Esfahani

**Affiliations:** 1 Department of Molecular Biotechnology at Cell Science Research Center, Royan Institute for Biotechnology, ACECR, Isfahan, Iran; 2 Department of Reproduction and Development at Reproductive Biomedicine Research Center, Royan Institute for Biotechnology, ACECR, Isfahan, Iran; 3 Isfahan Fertility and Infertility Center, Isfahan, Iran

**Keywords:** miRNA, Spermatogenesis, Male Infertility, Biomarker, Single Nucleotide Polymorphism

## Abstract

MicroRNAs (miRNAs) are small non-coding single stranded RNA molecules that are physiologically produced in eukaryotic cells to regulate or mostly down-regulate genes by pairing with their complementary base-sequence in related mRNA molecules in the cytoplasm. It has been reported that other than its function in many physiological cell processes, dysregulation of miRNAs plays a role in the development of many diseases. In this short review, the association between miRNAs and some male reproductive disorders is surveyed. Male factor Infertility is a devastating problem from which a notable percentage of couples suffer. However, the molecular mechanism of many infertility disorders has not been clearly elucidated. Since miRNAs have an important role in numerous biological cell processes and cellular dysfunctions, it is of interest to review the related literature on the role of miRNAs in the male reproductive organs. Aberrant expression of specific miRNAs is associated with certain male reproductive dysfunctions. For this reason, assessment of expression of such miRNAs may serve as a suitable molecular biomarker for diagnosis of those male infertility disorders. The presence of a single nucleotide polymorphism (SNP) at the miRNAs’ binding site in its targeted mRNA has been reported to have an association with idiopathic male infertility. Also, a relation with male infertility has been shown with SNP in the genes of the factors necessary for miRNA biogenesis. Therefore, focusing on the role of miRNAs in male reproductive disorders can further elucidate the molecular mechanisms of male infertility and generate the potential for locating efficient biomarkers and therapeutic agents for these disorders.

## Introduction

**Fig 1:**
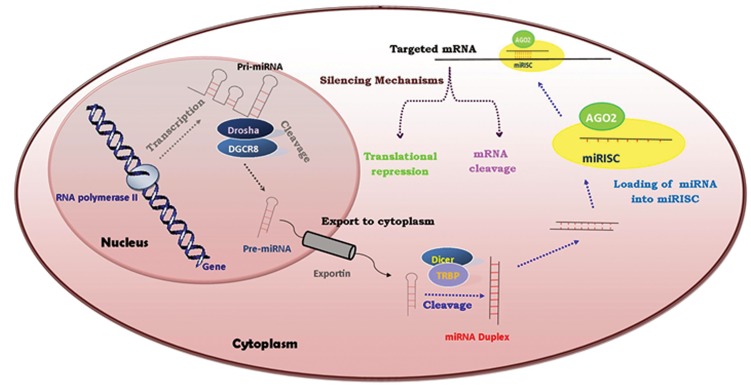
Biogenesis of miRNA: RNA polymerase II transcribes miRNA gene to produce Pri-miRNA having a stem loop structure. Drosha (a class 2 ribonuclease III) and its RNA-binding partner, DGCR8*, cut the stem loop structure to make pre-miRNA. Exportin 5 transports the pre-miRNA from the nucleus to cytoplasm. In the cytoplasm, Dicer (a RNase III endonuclease) and its RNA-binding partner, TRBP**, cleave pre-miRNA to generate a short double-stranded RNA molecule named miRNA duplex. One strand of this molecule is incorporated into the miRNA-induced silencing complex (miRISC) containig protein Argonaute-2 (AGO2) as a catalytic component to regulate the targeted mRNA. The mechanism of targeted mRNA silencing could be either translational repression or mRNA cleavage. miRNA; MicroRNA, * DGCR8; DiGeorge syndrome critical region gene and ** TRBP; Transactivating region binding protein.

MicroRNAs (miRNAs) are small non-coding single stranded RNA molecules of approximately 22 nucleotides that are physiologically produced in eukaryotic cells (1). After being expressed in the nucleus, miRNA plays a role in regulation of gene expression by pairing with its complementary base-sequence of the targeted mRNA molecule in the cytoplasm. This usually leads to gene silencing through degradation of targeted mRNA or interference with its translation (2, 3). After discovery of miRNAs, as single-stranded non-protein-coding regulatory RNA molecules in C. elegans by Lee et al. (4) in 1993, over 1500 miRNAs have currently been reported to be encoded by the human genome (5-7) which may target around 60% of mammalian genes in various human cell types (8). Figure 1 schematically depicts miRNA biogenesis.

Aside from the physiological regulation of gene expression in multiple biological processes such as cell cycle control and differentiation (9), cell growth and apoptosis (10) and embryo development (11), the role of miRNAs in development of many human diseases has been studied and reported. Mutation, dysfunction of biogenesis and dysregulation of miRNAs and their targets may lead to various diseases such as cardiovascular diseases (12), cancers (13), schizophrenia (14), renal function disorders (15), psoriasis (16), muscular disorders (17) and diabetes (18). A comprehensive database of whole miRNA-disease association, miRNA disease spectrum width (DSW), miRNA conservation and miRNA function has been published online at: http://202.38.126.151/hmdd/mirna/md/, entitled "The Human microRNA Disease Database". Among diseases, we have chosen to focus on male infertility with the intent to conduct a literature review of the role of miRNAs in this field.

Infertility is a problem in 10-15% of couples worldwide (19). Approximately, 50% of the infertility cases are attributed to male factors and 60-5% of these cases are idiopathic (20). Of note, the molecular mechanisms of many male infertility disorders are not clear (20). The aim of this review is to pursue the relation between miRNAs and male infertility.

### miRNA and spermatogenesis

The presence of miRNA in testis has been verified a few years ago (21). Later, researchers focused on finding the function of miRNAs in germinal tract physiology. Table 1 shows a brief description of the chronological order of discoveries related to miRNAs in testis molecular physiology [for additional information please refer to Papaioannou and Nef (22)].

**Table 1:** Discoveries related to microRNAs (miRNAs) in the male reproductive system


Discoveries	Method of analysis	Year	Reference

**Defining expression profiling of miRNA in human testis **	miRNA specific oligonucleotide microarrays	2004	21
**Report of Mirn122a as a down-regulator of germ cell transition protein 2 (Tnp2) messenger RNA**	Real-time, RT-PCR and ribonuclease protection assays	2005	23
**Defining of chromatoid bodies as intracellular nerve centers of themiRNA pathway in male germ cells**	-	2006	24
**In normal spermatogenesis, E2F1 mRNA translation is down regulated by miRNA (from the miR-17-92 cluster) to protect meiotic cells from apoptosis.Expression of miR-17-92 cluster is associated with reduced apoptosis in carcinoma in situ cells of the testis**	RT-PCR	2007	25
**Determination of miRNA expression profile in mouse testes in sexually immature and mature individuals**	Microarray assay andquantitative real-time PCR	2007	26
**Describing expression profiling of testis-expressed miRNAs to show some discriminatingly and uniquely expressed miRNA in murine testis**	Semi-quantitative RT-PCR analyses	2007	27
**Showing the differential pattern of gene expression for the Drosha, Dicer and Argonaute proteins in the germ and somatic cells of mouse testis**	Quantitative real-time PCR for mRNA of Drosha, Dicer and Argonaute proteins in the germ and somatic cells of mouse testis	2008	28
**Showing the localization of miRNAs (in addition to PIWI associated small RNA) in the nucleolus of Sertoli cells in addition to the chromosome cores, the telomeres and the XY body of spermatocytes**	Fluorescence in situ hybridization (FISH), microarray, quantitative real-time PCR	2008	29
**Dependency of proliferation of primordial germ cells and spermatogonia to miRNAs**	Real-time PCR using a specific knockout mice in which Dicer gene was solely absent in its germ cells	2008	30
**Dependency of spermatogenesis to Dicer in testis of mice**	Real-time quantitative PCR, microarray	2009	31
**Necessity of Drosha (RNase III) for spermatogenesis like Dicer**	Generation of postnatal male germ line-specific Drosha or Dicer knock-out mice	2012	32
**Identification of seven miRNA molecules discriminately expressed between postnatal gonocytes and spermatogonia to show their possible effect in maintaining germ cell differentiation and or pluripotency in mice**	miRNA microarray	2012	33


### Potential of extracellular miRNAs as biomarkers for infertility

In addition to the presence of miRNA at the cellular level, miRNAs have also been reported to be present in extracellular fluids such as plasma (34), saliva (35, 36), vaginal secretions, menstrual blood and semen (36). Aberrant expression of extracellular miRNA is attributed to different disorders (37). Altered expression of miRNAs, as with altered expression of some testis-specific mRNAs (38-41) has been recently reported in male infertility disorders, hence the possibility of locating potential miRNA biomarkers in male infertility has been proposed.

The first report of an alteration of miRNA expression in the testis of patients with non-obstructive azoospermia (NOA) has been reported by Lian et al. (42). Of note, that NOA which has a testis-origin, presents a diverse range of defects from hypospermatogenesis and sperm maturation arrest to Sertoli-cell-only-syndrome. Hence, assessment of differential expressions of miRNAs in these infertile individuals is a prerequisite.

Wang et al. (43) examined pooled semen samples obtained from infertile men and compared the results with normal fertile individuals as controls. They found alterations in miRNA profiles by Solexa Sequencing (a sequencing method based on reversible dye-terminators technology and engineered polymerases) in both azoospermia and asthenozoospermia. These authors considered a cut of value of 50-fold higher or lower expression as significant, which was further validated by real time RT-qPCR for 7 miRNAs (miR-34c-5p, miR-122, miR-146b-5p, miR-181a, miR-374b, miR-509-5p, and miR-513a-5p). The level of these 7 miRNAs was significantly lower in azoospermia and higher in asthenozoospermia compared to the control. Another noticeable finding of these authors was the stability of the above miRNAs to different conditions. They attributed this phenomenon to the small size of the miRNAs and their ability to bind with complex organic molecules in cell-free semen samples. Finally, they proposed that these 7 miRNAs might have confirmative molecular diagnostic value for male infertility.

In this regard, Wu et al. (44) evaluated the pattern of expression related to miR-I9b and let-7a in idiopathic infertile individuals with NOA or oligozoospermia by quantitative RT-PCR. They showed that these two miRNAs distinctively expressed at higher levels in infertile cases compared with fertile individuals. Consequently, they concluded that miR-I9b and let-7a were good diagnostic molecular biomarkers for idiopathic infertile cases with NOA or oligozoospermia.

### Single nucleotide polymorphism (SNP) and function of miRNA in spermatogenesis

Considering the diverse role of miRNA in spermatogenesis, therefore any polymorphism in related genes might lead to infertility. Zhang et al. (45) were the first researchers who reported an association between miRNA-binding site single nucleotide polymorphism (SNP) and idiopathic male infertility. They systematically surveyed all SNPs in the 3´ UTR of 140 mammal spermatogenesis-related genes and observed that some SNPs within miRNA-binding sites might be related to idiopathic male infertility.

Among 140 surveyed genes, 6 SNPs (two in CYP19, Serpina5 and four in CGA, CPEB1, and CPEB2), involved in down regulation of gene expression in spermatogenesis and meiosis and were predicted to have possibility for altering the binding affinity of miRNA by using bioinformatically specialized algorithms (Pictar, miRanda, Targetscan, and RNAhybrid). The effectiveness of these SNPs in male infertility was analyzed in a case-control study using PCR followed by restriction digestion of the amplified fragments for genotyping. They realized that T substituted for A in rs6631 which lead to diminished binding ability of miR-1302 to its binding site in CGA *in vitro*, with a subsequent overexpression of CGA. Therefore an association between this special SNP and male infertility was concluded.

Recently Qin et al. (46) evaluated the relation between seven SNPs in the genes of Drosha (rs10719, rs2291109, rs17409893 and rs642321) and Dicer (rs13078, rs1057035 and rs12323635) and infertility. Performing genotyping by means of real time PCR, these researchers showed that SNPs in rs10719, rs12323635 and rs642321 were related with male infertility in the examined Han Chinese population.

## Conclusion

Considering limited number of studies performed in this field, little has been clarified about miRNA-mediated gene regulation in spermatogenesis. In addition, there are few numbers of published studies that focus on the role of miRNAs in male infertility. This review has aimed to elaborate the role of miRNA in the male reproductive system. Any disorder or failure in this system can result in male infertility. Therefore, a dysfunction in miRNA processing as with Dorsha and Dicer can result in azoospermia and infertility. Other anomalies such as dysregulation in expression of certain miRNAs or SNP in the miRNA binding site or SNP in the genes involved in biogenesis of miRNA can be related to infertility. Hence, in this regard, scientists suggest that assessment of miRNA may have future diagnostic value and shed more light on the molecular mechanisms of male infertility. Thus, new paths can be opened for future treatments of male infertility or even in the design of new contraceptive drugs.
